# Evaluation of the predictive value of the body-mass-index choosing perforator flaps from different donor sites

**DOI:** 10.1186/s12893-023-01962-3

**Published:** 2023-03-27

**Authors:** Tobias R. Mett, Stephanie Boetger-Bolten, Florian Bucher, Peter M. Vogt

**Affiliations:** 1 Department of Plastic, Aesthetic, Hand and Reconstructive Surgery, Burn Center, Hannover Medical School, Carl-Neuberg-Str.1, 30625 Hannover, Germany; 2 Present address: Department of Plastic, Aesthetic, and Reconstructive Surgery, Evangelical Hospital Göttingen-Weende, An Der Lutter 24, 37075 Göttingen, Germany

**Keywords:** Microsurgery, Free flap, BMI, Body mass index, Flap thickness

## Abstract

**Background:**

Free flap design must fulfill different criteria to ensure functional and aesthetic reconstruction of different types of defects in different body areas. A four-dimensional planning concept was used including flap length, width, thickness and tissue composition. This study evaluates if body-mass-index (BMI) has a predictive value for flap design.

**Methods:**

A prospective study including hospitalized patients in the Department of Plastic, Aesthetic, Hand and Reconstructive Surgery was conducted. Not taking into account the reason for admission, the patients were examined based on age, weight, height, BMI and sex. The areas of a potential harvest of free fasciocutaneous and perforator flaps were measured using ultrasound examination to determine the thickness of the subcutaneous layers and prove blood perfusion.

**Results:**

Over the period of four months, a total of 101 patients (36 females and 65 males) were included in this study and gave written consent. No statistical significance regarding the demographic data such as age, sex and BMI could be identified. An average to high correlation between free flap thickness and BMI was shown for the thoracodorsal artery perforator (TDAP), anterior lateral thigh (ALT) and deep inferior epigastric perforator (DIEP) flap in both, male and female patients.

Free flaps of distal body parts such as the interosseous posterior flap, showed a lower correlation. No correlation using the Pearson coefficient could be found for age and volume.

**Conclusion:**

Our study demonstrated that the BMI is a predictive indicator that can be used in the preoperative planning of reconstructions using free flaps. Depending on the defect location, the BMI can be considered to predict the thickness of the free flap and may influence the surgeon’s choice.

On the other hand, a lower correlation between BMI and flap thickness encourages the use of standard flaps if more volume is desired, as the DIEP flap might be sufficient even in normal-weight women. Flaps of distal body parts, such as the forearm or lower leg, are not prone to such predictions and require other selection criteria.

**Supplementary Information:**

The online version contains supplementary material available at 10.1186/s12893-023-01962-3.

## Background

Free flap requirements include reliable microvascular anatomy, matching tissue types at the defect area and low donor site morbidity as well as a growing desire for an aesthetic appearance after defect coverage [1, 6].

Nowadays, perforator flaps are routinely used by plastic surgeons for reconstructive coverage of defects of various sizes from head to toe. Considering the aesthetic goal, it is mandatory to replace like with like. Therefore the preoperative flap design should include a four-dimensional evaluation of the donor site [1]. Important free flap parameters regarding the dimensions include length, width and thickness. Moreover, the fourth dimension is the composition of different tissues, such as fat, fascia and muscle tissue, and the perfusion pattern of each. Usually, the most demanding defects require relatively thin transplants to meet the aesthetic aspect; for example, at the scalp, face, hand or lower leg and foot [2–7]. As a result, free fasciocutaneous flaps can be harvested from different body sites depending on the individual body constitution [2].

It seems to be obvious, that body mass index (BMI) and flap parameters may correlate, but to the best of our knowledge, no study regarding the flap thickness in adipose patients could be identified. Since the number of overweight people and correlating morbidities is endemic and rising, it is important to be able to estimate flap characteristics for preoperative planning [8]. In patients with high BMIs, preoperative diagnostics might be biased, in terms of accuracy. It has been proven that the specificity of hand doppler decreases with rising BMI [9]. This should be taken into account in patients examined using ultrasound-based modalities. The results of this study should facilitate preoperative flap choice and planning for an optimal reconstructive and aesthetic outcome.

## Methods

### Patients

A prospective study on hospitalized patients in the Department of Plastic, Aesthetic, Hand and Reconstructive Surgery was conducted.

The study was approved by the clinical ethics committee of the Hannover Medical School (No. 3163–2016). Data collection was performed anonymously using a digital clinical patient database.

The subjects were visited by the researchers during their hospital stay and gave informed consent regarding the use of their patient data and intraoperative photographs.

The study was performed in conformity with the provisions of the Helsinki Declaration (revised in 2013).

Patients aged above 18 years were included. Exclusion criteria were patients under the age of 18 years, patients with surgical wounds in the flap donor areas and patients for whom a painless examination could not be guaranteed. In addition, patients requiring intensive care monitoring or isolation were excluded from the study. A total of 101 subjects were included in this study.

A written explanation and informed consent for the study were part of the preoperative education. After the patients were informed regarding the course of the examination and agreed to participate in the study, the investigation was carried out in the patient's room under a room temperature of 69.8 – 73.4 °F (21–23 °C) and without physical exercise.

The investigation included a collection of patient data on a self-designed form, including age and gender, height and weight.

BMI Nicotine abuse, intake of oral anticoagulation and the presence of a peripheral arterial disease (PAD).

The thickness of subcutaneous fat was measured on a total of 11 specified areas of the body by ultrasound. Potential donor sides were evaluated for a potential flap harvest. The chosen flaps were the lateral upper arm flap, the posterior interosseous flap, thoracodorsal artery perforator flap (TDAP), deep inferior epigastric perforator flap (DIEP), anterior lateral thigh flap (ALT) and dorsal pedis flap. The changes in the tissue composition in different body areas were covered by the examination of the upper and lower extremities and the trunk. For the radiological examination, an ultrasound device was used with the following image methods: B-image method (brightness modulation), color-coded duplex sonography (CCDS) method (color-coded duplex sonography) and power Doppler technique to visualize the vascularity of the selected area. (Diagnostic Ultrasound System: TOSHIBA, XARIO V 8.0: 1–1, Shibaura 1-chome, Minato-ku, Tokyo 105–8001, Japan).

Two probes were used: PLT-805AT, scan type: linear 58 mm, frequency: 8.0 MHz; B-mode frequency:12.0/10.0/8.6/6.2/ 5.0/ 9.0/ 8.4/ 7.6/ 7.2/ 6.6 MHz; and PLT-120BT, scan type: Linear 38 mm, frequency 12.0 MHz, B-mode frequency 14.0/ 12.0/ 9.3/ 8.0/ 7.2/ 14.0/ 12.0/ 10.0/ 8.0/7.0 MHz.

### Examination course

The examination time of each patient ranged from 30 to 60 min.

Patients were examined in their wards in their beds in a supine position, or sitting position for the dorsal body regions. A sonographic determination of the thickness of the subcutis was performed for selected donor site areas of the body. The distance from the skin surface to the muscle fascia was determined in each case by the same investigator. For a reliable determination of the muscle location, the patient was asked to contract the underlying muscle in the examined area. To avoid artifacts, care was taken to keep the legs in neutral zero position. Vascular perforators were identified using power and color doppler.

### Statistical tools

Microsoft Office Excel 2010 (Microsoft Corp, Redmond, WA) was used for data collection as well as for statistical analysis. P-values < 0.05 were considered statistically significant. The relations of collected data have been analyzed using the Pearson correlation coefficient referring as r with values from 0 to > 0.9. Interpretation followed the classification:

- 0.0—0.2, very low correlation
- 0.3—0.5, low correlation
- 0.6—0.7, average correlation
- 0.9—0.9, high correlation
-  > 0.9, very high correlation

## Results

A total of 101 patients (65 males, 36 females) were included. The age of the male subjects ranged from 18 to 85 years, with a mean value of 44 years. The age of the female subjects was between 18 – 86 years, with a mean of 47 years.

**Fig. 1:**
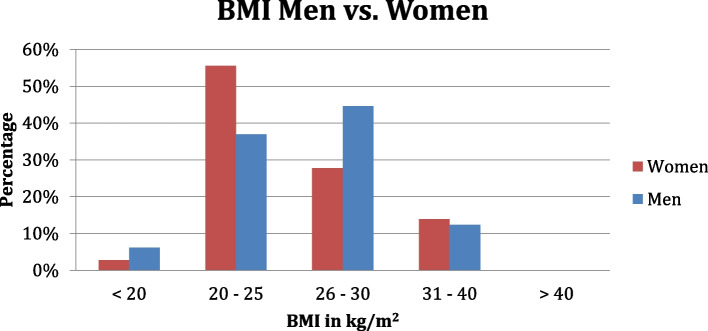
Distribution of the BMI in kg/ m^2^ between women and men

The BMI was calculated according to the following formula: BMI = body weight (in kg)/ (height in m)^2^. The mean value was 26 kg/m^2^ for the men and 25 kg/m^2^ for the women, without statistically significant differences. The male and female test subjects each had a maximum BMI of 39 kg/m^2^. The minimum BMI was 17 kg/m^2^ for men and 18 kg/m^2^ for females. Of the females participating in this study, the BMI of 19 females (53%) was within a normal range (18-25 kg/mm^2^). For 17 subjects (47%) the BMI was outside the normal range, of which one subject (3%) was below the normal BMI and 16 subjects (44%) were above the normal BMI. Regarding the males, 24 subjects (37%) had a BMI within the normal range. For 41 subjects (63%) the BMI was outside of this range. 37 subjects (57%) had a BMI higher and four subjects (6%) were below the normal range (Fig. 1).

The distribution of BMI between men and women was relatively balanced. Among the females, the proportion of patients within the range of normal weight was higher compared to males.

The collected data including BMI was correlated with the thickness of subcutaneous fat using the Pearson correlation coefficient. The correlations between the subcutaneous layer of adipose tissue and the BMI are shown in Table 1.

**Table 1:** Correlations between the subcutaneous layer of adipose tissue in distinct flap donor sites and the BMI

Women	Men
< 0.2 (very low correlation): • none	< 0.2 (very low correlation): • none
0.3—0.5 (low correlation): • posterior interosseous flap • lateral upper arm flap	0.3—0.5 (low correlation): • dorsal pedis flap • posterior interosseus flap
0.6—0.7 (avarage correlation): • dorsal pedis flap • DIEP flap	0.6—0.7 (avarage correlation): • ALT flap • lateral upper arm flap • TDAP flap
0.8—0.9 (high correlation) • ALT flap • TDAP flap	0.8—0.9 (high correlation) • DIEP flap
> 0.9 (very high correlation) • none	> 0.9 (very high correlation) • none

*Abbreviations: ALT* anterior lateral thigh, *DIEP* deep inferior epigastric perforator flap, *TDAP* thoracodorsal artery perforator flap

**Fig. 2:**
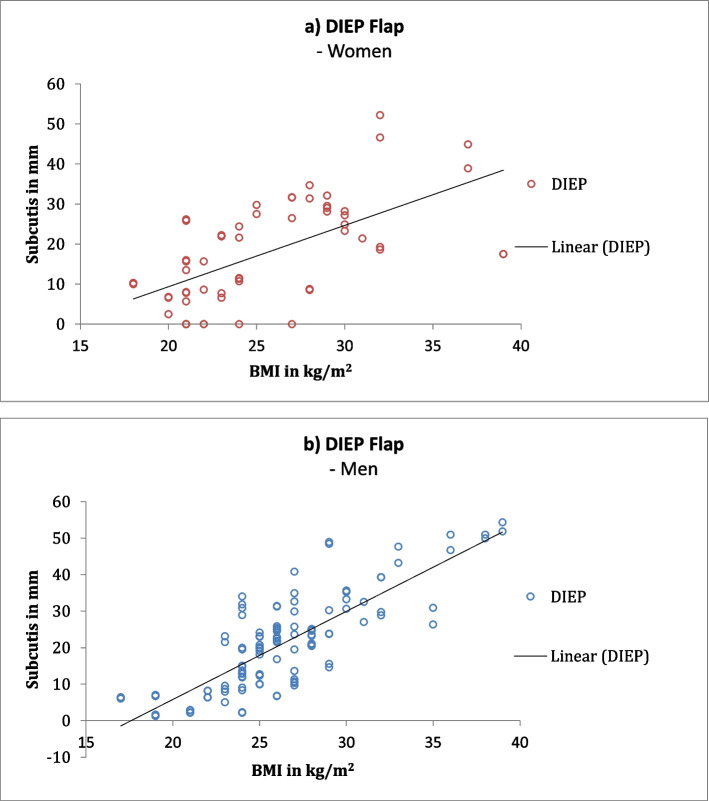
DIEP flap: Correlation coefficient between subcutaneous fat and BMI showing an average correlation for women (**a**) and high correlation for men (**b**)

**Fig. 3:**
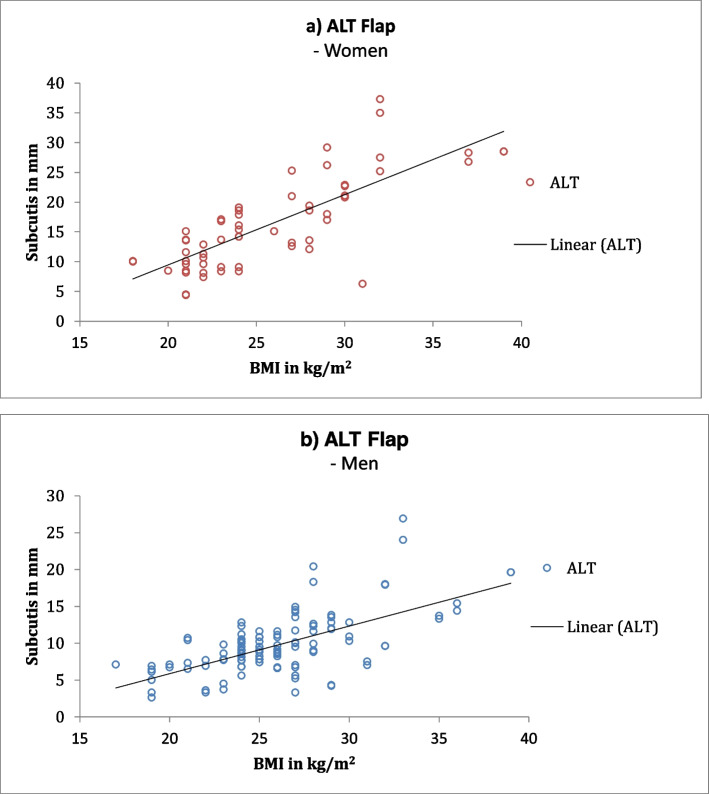
ALT flap: Correlation coefficient between subcutaneous fat and BMI showing a high correlation for women (**a**) and average correlation for men (**b**)

**Fig. 4:**
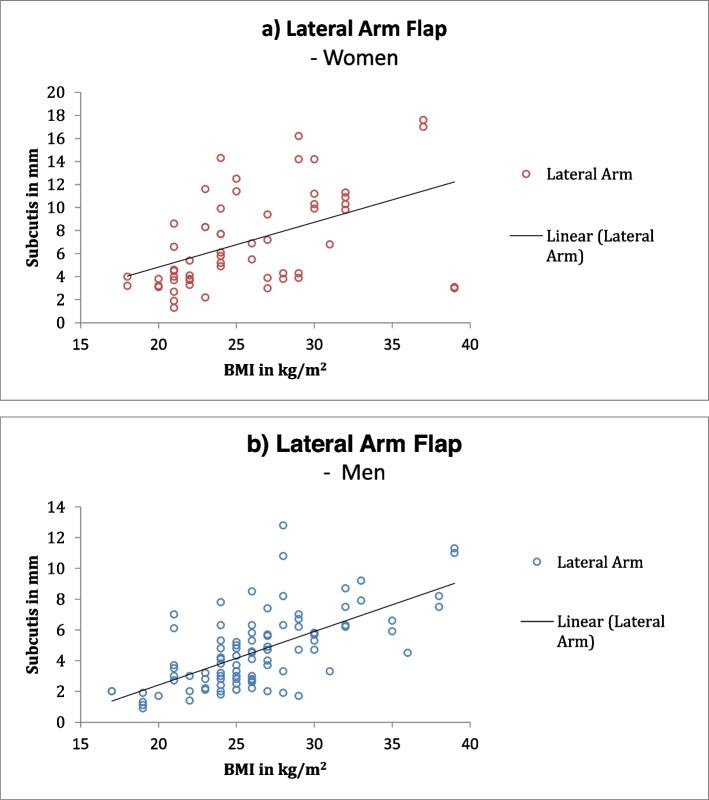
Lateral arm flap: Correlation coefficient between subcutaneous fat and BMI showing a low correlation for women (**a**) and average correlation for men (**b**)

Gender differences were identified using correlation coefficients. The lateral arm flap showed a higher correlation in men. The dorsalis pedis flap, on the other hand, showed a higher correlation among women. The DIEP flap had the highest correlation among men, whereas among women the ALT and TDAP flaps had the highest correlation (Fig. 2 DIEP, Fig. 3 ALT, Fig. 4 lateral arm, Fig. S1 TDAP, Fig. S2 posterior interosseus, Fig. S3 dorsalis pedis).

Patient age and the characteristics of the subcutaneous fat layer showed significant correlations which are presented in Table S1. These correlations showed that between the selected variables in women and men, only little or no correlation at all could be found. The men only had a low correlation at the location of the dorsalis pedis flap. In the case of all other flaps the correlation coefficients were below 0.2, and sometimes even negative.

During the study, 11 patients have been taken to the OR for free flap surgery. ALT flaps for lower extremity reconstruction have been carried out in 6 patients. DIEP flaps have been performed for breast reconstruction in 4 patients and one forearm flap was used to cover a facial defect. Due to the study’s concept, detailed information is present from only one patient receiving a DIEP flap. A 34-year-old woman suffering from chronic mastitis in the left breast and presenting with tuberous breasts underwent a subcutaneous mastectomy and immediate DIEP Flap reconstruction for the left breast. Due to a BMI of 35 kg/m^2^ a high-volume flap could be expected. Furthermore, an additional fat harvest from excessive tissue could be taken for free fat grafting to address the tuberous breast deformity (Fig. S4 and S5).

## Discussion

This study compared the subcutaneous layer thickness of the perforator and fasciocutaneous flaps in correlation to the BMI, age and gender of Caucasian patients. The investigations resulted in important answers to the following questions:

Does a relationship exist between the subcutaneous layer thickness of certain areas of the body and the BMI, age and gender? This information is relevant for the preoperative planning of free flaps. The thickness of the subcutaneous layer plays a decisive role in the function and aesthetics of a flap and may change the preoperative plan [10]. The more the surgeon knows about flap characteristics, the better the patient’s information and the final operation can be planned. Moreover, correlations to potential complications can be made, respecting, that higher BMIs may lead to a higher complication rate in free flap surgery [11]. This may be explained by the thick flaps with more fatty tissue which might promote wound healing disturbances due to decreased perfusion patterns.

Fasciocutaneous flaps gained increasing popularity as working horses in plastic surgery [5, 12, 13]. Flap dissection depends on the local blood supply and the tissue quality but is usually facilitated by the knowledge of tissue layers and vascularity characteristics [1, 13, 14]. Thin fasciocutaneous flaps with constant anatomy have the ability to make a successful free flap surgery very likely. In particular, thick layers of fat are very bulky and are accompanied by wound healing disturbances, as adipose tissue has a poor blood supply. In this respect, the success of fasciocutaneous flap reconstructive surgery depends on the body area, flap availability and patient characteristics. Patients of Caucasian origin have an increasing BMI [8, 15]. This study seeks to predict the match of subcutaneous layer thickness and flap thickness in different body regions by means of BMI, which can be easily calculated during everyday practice using just patient height and weight. 5 perforator flaps (DIEP, ALT, TDAP, lat. arm, post. interosseus) and one axial pattern flap (dorsalis pedis flap) have been chosen to represent the trunk and most distal parts of the body, although the perfusion patterns differ between that choice. To the authors’ best knowledge, this is the first study that established a correlation between the subcutaneous adipose tissue layer of a desired flap and BMI.

According to a study by the Federal Statistical Office in 2017, the BMI of the German population is distributed as follows: underweight: 2%, normal weight: 45.3%, overweight: 36.4% and obesity: 16.3%. Massive obesity is not considered separately in this study [8, 15]. The subjects examined by our study represented a surprisingly comparable cross-section through the different ranges of BMI with an almost identical distribution: underweight: 3%, normal weight: 47%, overweight 37% and obesity: 13%. All of the examined flaps had at least a low correlation between BMI and the subcutis. There was a high correlation among women and the ALT and TDAP flaps showed a correlation among men in just the average range. In men, the DIEP flap showed the highest level of correlation, which, interestingly, was in the middle range for women. The reasons might be the different fat distribution and postpartum situations, which were not examined in detail. Even women with normal BMI may present with a thicker fat layer in the lower abdominal region in comparison to normal-weighted men. This could be due to gender-specific differences in body shape. While for men, increased fat accumulation in the abdominal region is typical—an android body shape (trunk-emphasized, visceral, central), the so-called "apple shape"—women tend to show an increased accumulation of fat in the thighs, hips and buttocks, which is the gynoid (hip-accentuated, peripheral) fat distribution of the so-called "pear shape" [16]. As a result, a DIEP flap with proper volume might be expected even in women with normal BMIs. Therefore, the average correlation of the BMI is not an exclusion criterion for a DIEP flap in normal-weighted women when planning, for example a breast reconstruction. Nevertheless, thick DIEP flaps are almost guaranteed in women with increased BMI. This information allows preoperative assumption of the needed flap size and enables a salvage of flap excesses (Fig. S4 and S5).

A study by Wenzel in 2003 confirmed that the BMI is an optimal assessment measure for the determination of body fat mass [17]. According to Wenzel et al., BMI is statistically significant and highly correlated with the amount of subcutaneous adipose tissue and body fat mass [17]. Specific body areas were not differentiated by the authors.

Nevertheless, the BMI is internationally recognized as a standard measurement tool, even though it may vary due to different fat distribution patterns in different ethnic groups and depends on the fitness level of each patient. As an indirect measurement of the body fat percentage, the BMI is considered to be the best in relative terms anthropometric measure [15].

Our results suggest that certain areas of the body (upper thigh = ALT, torso = TDAP, abdomen = DIEP) correlate with the calculated BMI which determines the thickness of the subcutis, and thus, can predict the graft thickness of a flap. This examination showed that the grafts in the shoulder region generally have a thin subcutis; however, a correlation to the patient’s BMI still exists. Many plastic surgeons took advantage of this during the last four decades. [18–21]. Even for the reconstruction of head and neck defects, with a higher demand for thin and suitable transplants, these flaps are well established [3]. The present study found a correlation between the BMI in both sexes. This finding also applies to the thigh, as the ALT flap is generally used as a relatively thin transplant, but it is still correlated to the patient’s BMI [14].

Our findings proved that the above-mentioned correlation decreases from proximal to distal body regions.

The choice of a flap used for reconstruction can be made according to the following criteria: extension of the defect, required length of the pedicle, skin properties, position of the patient for the flap harvest/ inset and flap thickness. For the latter, the BMI allows an estimation.

Regarding the results, the surgeon might avoid flaps with high correlations to the BMI if a thin fasciocutaneous flap is needed. For example, an ALT flap in women (CC < 0.9) might be replaced by a lateral upper arm flap (CC < 0.5) with its very constant vascular system of the subscapular artery as an excellent alternative. This region should therefore be considered when BMI is high and alternatives are needed [7].

In contrast, a statistically significant correlation between the subcutaneous fat layer and age could not be identified by our study. For both sexes, all values of the correlation coefficients were below 0.2, which corresponds to a very low correlation. One exception was found in the male population for the dorsalis pedis flap with values just over 0.2, representing a low correlation. In some cases, negative values were shown, indicating no correlation at all. These results show that compared to the correlation of the subcutaneous tissue and the BMI, the patient’s age is not statistically significantly correlated to flap characteristics. There are some flaws in this study that should be mentioned. Since the study only included Caucasian individuals by chance, no statement could be made about different fat distribution patterns in different ethnicities. Only some of the patients (11/101) did refer to the OR for free flap surgery during the study’s timeframe. Therefore, a clinical correlation and proof of concept could not be made, as the study design did not aim to investigate intraoperative findings.

Even though superthin flaps with suprafacial harvest emerged as advancing techniques in microsurgery the present study did not differ between superficial and deep fat compartment as this technique was not standardized in clinical routine at the time of data collection. These aspects should be addressed in continuative research. The findings of this study are now implemented in daily routine when educating younger surgeons and patients.

## Conclusion

This study investigated potential correlations of the subcutaneous layer thickness in perforator and fasciocutaneous flaps with BMI, age and gender for a Caucasian patient population. The results suggest that in certain areas of the body, a statistically significant correlation between BMI and the subcutaneous fat layer exists, indicating the thickness of the subcutaneous fat can be predicted. Especially for the ALT, TDAP, and DIEP flaps, a prognosis is possible, according to our findings. For the other investigated body areas, there exists at least a medium to lower predictability. In contrast, age cannot be used as an indicator of flap design since almost all cases showed a low or no correlation. Gender-specific differences have been found in the areas of the TDAP, ALT and DIEP flap. A lower correlation coefficient should raise the surgeon’s suspicion about whether the distinct flap might meet a patient’s expectations for various BMIs. A high BMI might lead to an alternative donor site when a thin flap design is required. On the other hand, a low BMI does not need to be an exclusion criterion if a certain volume is required, as it is useful for preoperative planning in breast reconstruction (e.g., DIEP flaps). Even in situations of oversized flaps a flap salvage for other reconstructive techniques can be assumed by knowledge of the amount of subcutaneous fat.

## Supplementary Information

**Additional file 1: Fig. S1.** TDAP flap: Correlation coefficient between subcutaneous fat and BMI showing a high correlation for women (a) and average correlation for men (b). **Fig. S2.** Posterior interosseus flap: Correlation coefficient between subcutaneous fat and BMI showing a low correlation for women (a) and men (b). **Fig. S3.** Dorsalis pedis flap: Correlation coefficient between subcutaneous fat and BMI showing an average correlation for women (a) and low correlation for men (b). **Fig. S4.** DIEP Flap thickness of 5cm measured after harvest of a hemi DIEP flap of an adipose, female patient for breast reconstruction with a BMI 35kg/m2. **Fig. S5.** Salvage of flap excess after harvest of a hemi-DIEP Flap from an adipose, female patient for breast reconstruction with a BMI 35kg/m2. Lipoaspiration for autologous lipografting for the contralateral breast for and a final contouring. **Table S1.** Correlations between the subcutaneous layer of adipose tissue in distinct flap donor sites and the patient’s age.

## Data Availability

A dataset of raw data was deposited on www.synapse.org, ID syn33569299.

## Abbreviations

°C: Degree Celcius
°F: Degree Fahrenheit
ALT: Anterior lateral thigh
B-mode: Brightness mode
BMI: Body mass index
CC: Correlation coefficient
CCDS: Color-coded duplex sonography
DIEP: Deep inferior epigastric perforator
Fig.: Figure
kg: Kilogram
lat.: Lateral
m: Meter
Mhz: Megahertz
No.: Number
PAD: Peripheral arterial disease
Post.: Posterior
TDAP: Thoracic dorsal artery perforator
y: Years
