# Supplementary material for: Evaluation of the predictive value of the body-mass-index choosing perforator flaps from different donor sites
Source: BMC Surg. 2023 Mar 27;23:65. doi: 10.1186/s12893-023-01962-3 (PMC10041734; doi:10.1186/s12893-023-01962-3)
Supplement: Supplementary file 1 — Additional file 1: Fig. S1. TDAP flap: Correlation coefficient between subcutaneous fat and BMI showing a high correlation for women (a) and average correlation for men (b). Fig. S2. Posterior interosseus flap: Correlation coefficient between subcutaneous fat and BMI showing a low correlation for women (a) and men (b). Fig. S3. Dorsalis pedis flap: Correlation coefficient between subcutaneous fat and BMI showing an average correlation for women (a) and low correlation for men (b). Fig. S4. DIEP Flap thickness of 5cm measured after harvest of a hemi DIEP flap of an adipose, female patient for breast reconstruction with a BMI 35kg/m2. Fig. S5. Salvage of flap excess after harvest of a hemi-DIEP Flap from an adipose, female patient for breast reconstruction with a BMI 35kg/m2. Lipoaspiration for autologous lipografting for the contralateral breast for and a final contouring. Table S1. Correlations between the subcutaneous layer of adipose tissue in distinct flap donor sites and the patient’s age. [file 12893_2023_1962_MOESM1_ESM.docx]

**Supplements**

Fig. S1 TDAP flap: Correlation coefficient between subcutaneous fat and BMI showing a high correlation for women (a) and average correlation for men (b)

Fig. S2 Posterior interosseus flap: Correlation coefficient between subcutaneous fat and BMI showing a low correlation for women (a) and men (b)

Fig. S3 Dorsalis pedis flap: Correlation coefficient between subcutaneous fat and BMI showing an average correlation for women (a) and low correlation for men (b)


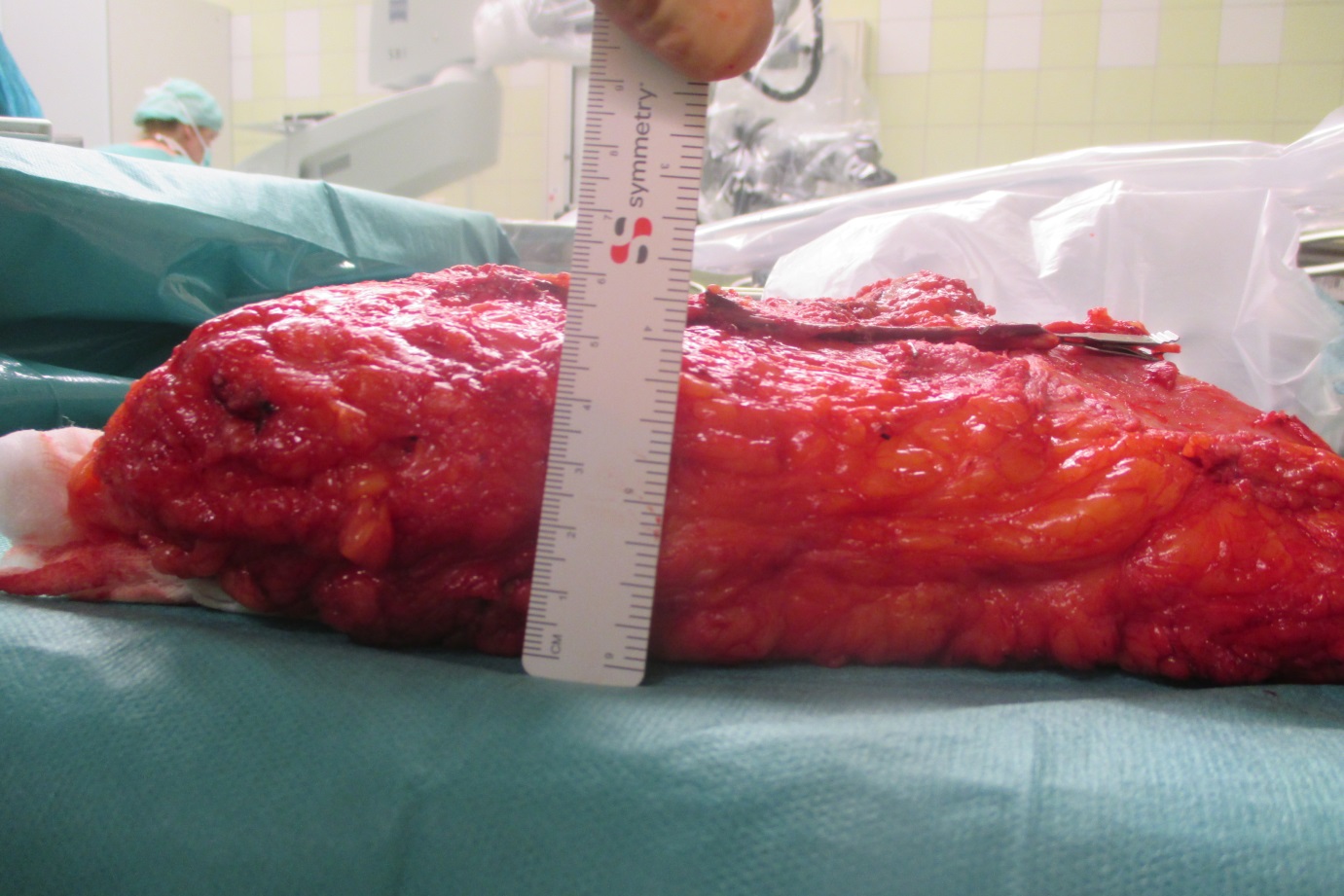


Fig. S4: DIEP Flap thickness of 5cm measured after harvest of a hemi DIEP flap from an adipose, female patient for breast reconstruction with a BMI 35kg/m^2^


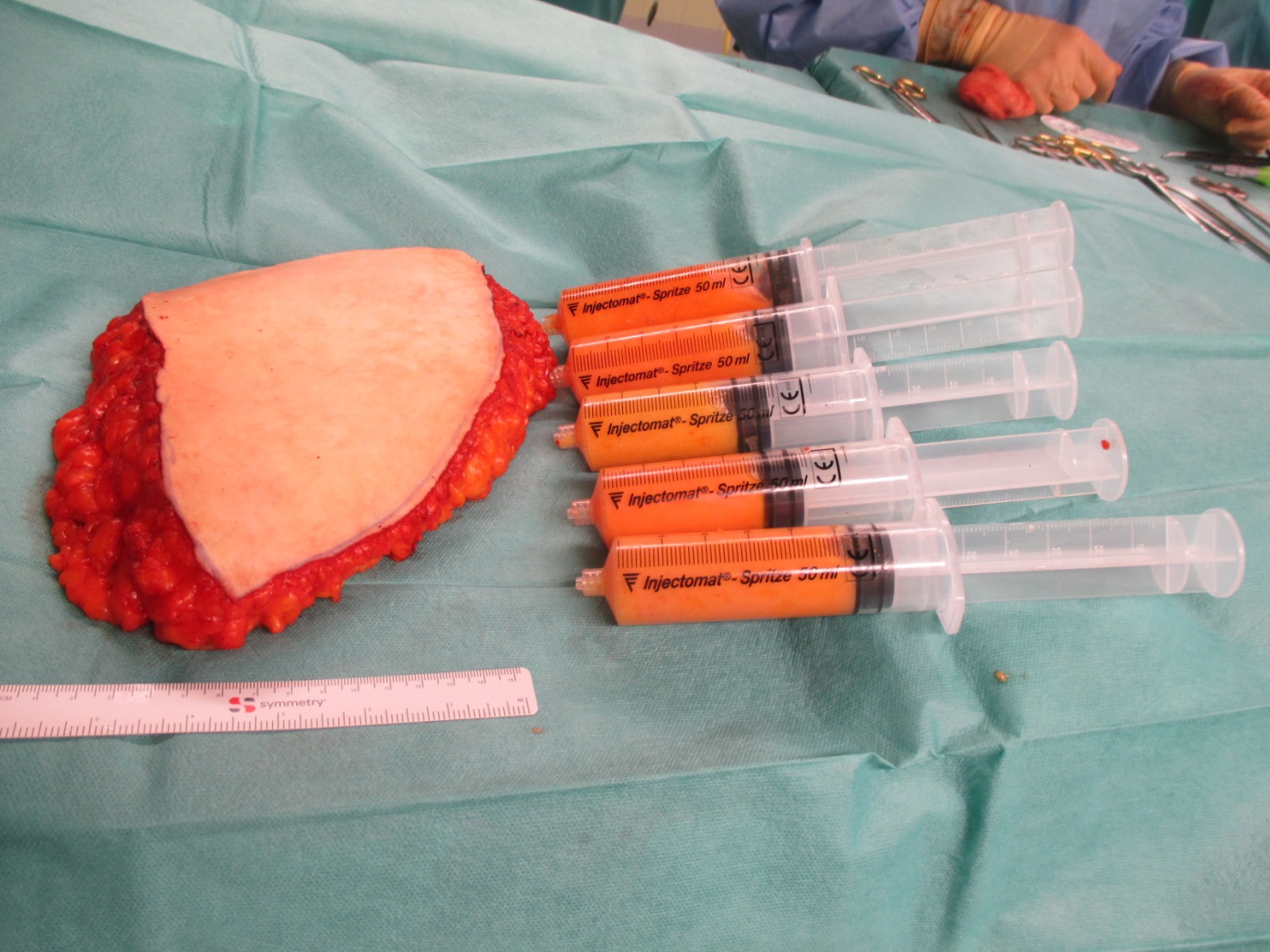


Fig. S5: Salvage of flap excess after harvest of a hemi-DIEP Flap from an adipose, female patient for breast reconstruction with a BMI 35kg/m^2^. Lipoaspiration for autologous lipografting for the contralateral breast for and a final contouring.

| **Women** | **Men** |
| --- | --- |
| < 0.2 (very low correlation):   - dorsal pedis flap - ALT flap - DIEP flap - TDAP flap - posterior interosseous flap - lateral upper arm flap | < 0.2 (very low correlation):   - ALT flap - DIEP flap - TDAP flap - posterior interosseous flap - lateral upper arm flap |
| 0.3 - 0.5 (low correlation):   - none   lateral upper arm flap | 0.3 - 0.5 (low correlation):   - dorsal pedis flap   posterior interosseus flap |
| 0.6 - 0.7 (avarage correlation):   - none | 0.6 - 0.7 (avarage correlation):   - none |
| 0.8 - 0.9 (high correlation)   - none | 0.8 - 0.9 (high correlation)   - none |
| > 0.9 (very high correlation)   - none | > 0.9 (very high correlation)   - none |

Table S1: Correlations between the subcutaneous layer of adipose tissue in distinct flap donor sites and the patient’s age
